# Women, Culture and Africa’s Land Reform Agenda

**DOI:** 10.3389/fpsyg.2018.02234

**Published:** 2018-11-23

**Authors:** Adeoye O. Akinola

**Affiliations:** Public Administration, University of Zululand, Richards Bay, South Africa

**Keywords:** culture, women rights, land reform, land labor, human capacity building, Africa

## Abstract

Pre-colonial Africa prides itself on adherence to diverse cultural affinity and traditional belief systems, which defines the place of women in respect to land access, use and ownership. Land resources continue to play important roles in both agrarian and industrial societies; thus the absence of effective land management and gender construction in land allocations has deepened gender inequality, restricted women’s capacity building and agricultural development in Africa. This article explores the impact of traditional African practices and cultural beliefs on women’s land ownership and use, and also reconciles women’s land rights (access and control) with the realities of land reform in post-colonial Africa. It explores how gender inequalities, in terms of land ownership and rights, have jeopardized attempts at agricultural productivity and sustainable development in Africa. However, it is tasking to ‘universalize’ African culture and locate it in a center, due to the diverse cultural values found in Africa. However, there are certain belief systems that run through most African communities, such as the denial of women’s land rights and the patriarchal nature of societies. Thus, the article found that, despite the development of legal frameworks that expand women’s property rights, cases of cultural impediments to the exercise of land rights abound in Africa.

## Introduction

Discourses on land resources, land conflict and land reform^1^ have dominated Africa from the late 1970s. From South Africa to Zimbabwe, and Congo DRC to Nigeria, land related issues have confronted African states and threatened their territorial integrity. Even in northern Africa, Egypt and Ethiopia have been at loggerheads over land-related politics. Land constitutes a productive asset and is a major source of capital for the poor (Lipton, 2009). Indeed, in rural and even urban centers, land is wealth. The importance of land and its unequal distribution, between male and female, has called for a rethinking of land reform and women’s land rights^2^ in Africa. Despite the enactment of gender-free laws in many African states, women have been consistently denied access to land in many parts of the continent, particularly in the areas.

Generally, gender roles are determined by ideological, ethno-religious, economic and socio-cultural factors, which are major determinants of the distribution of responsibilities and resources between men and women (Food and Agriculture Organization [Fao], 2011). Men have assumed the custodianship of land for agricultural purposes. Women farmers are largely excluded from modern contract-farming arrangements because they have no tenure security, thereby curtailing agricultural productivity (Food and Agriculture Organization [Fao], 2011). There is compelling evidence which shows that women seem to have higher land productivity than men. How do we reconcile this and ensure land productivity? Advocating for the attainment of women’s land rights is synonymous with land productivity. Certainly, Africa has gone past the question, ‘Do women need independent land rights?’, and many states have also modified their legal framework to accommodate women’s land rights; however, the actualization of the rights has become disputed, especially among rural dwellers.

Land rights are usually conceived as the rights and legitimacy to access, use, own, control, enjoy and exploit land. In terms of gender construction, land rights go beyond merely the rights to use or control land as a vital economic asset, but also involve laying claim to information about, decision-making around (for instance, lease or sell) and ultimately enjoying the benefits thereof (Wanyeki 200). In South Africa for instance, despite the constitutional guarantee of gender equality, land reform in many rural areas has not benefitted women due to customary law practices which deny women access to land (Rangan and Gilmartin, 2002: p. 633). In Africa, the bulk of the land (about 75%), as an economic asset, is under customary tenure, administered by norms, historical practices and unwritten law based on tradition and cultural affiliation (Odeny, 2013: p. 8). The objectives of effective land reform are to redistribute wealth, achieve agrarian reform, increase access to land and bridge the gender-gap in the land sector.

Globally, “at least 1.5 b people today have some farmland as a result of land reform, and are less poor, as a result. But huge, inefficient land and land inequality remains, or have reemerged in low income countries” (Lipton, 2009: p. 8). Africa continues to play host to many low-income countries. Therefore, acquiring women’s property rights is crucial to the socio-economic development of the continent (Garvelink, 2012). The United Nations’ Millennium Development Goal 3, the promotion of gender equality and empowerment of women, highlights the imperativeness of implementing laws and policies that would abolish social-economic and political exclusions of women (United Nations Development Programme [UNDP], 2015). Global non-state actors, like the World Bank, Food and Agriculture Organization (FAO) of the United Nations and the Convention on the Elimination of Discrimination Against Women continue, to advocate for gender parity in the land sector.

These organizations emphasize the importance of land to the developmental agenda of developing countries. Land deprivation is connected with diminishing livelihoods and increasing food insecurity in the continent. From Southern to East, and West Africa, poverty reigns. Furthermore, the issue of skyrocketing food prices persists, while food shortage becomes disturbing. Like their male counterparts, a large percentage of women are active farmers and highly dependent on the agricultural sector for their livelihood. Similarly, Odeny commented thus,

“Land is one of the cornerstones of economic development on which farmers, pastoralists and other communities base their livelihoods. Land is also a significant component of business assets, which play significant role in business investment strategies. Thus, securing land rights can have a profound impact on economic development…land is a source of identity and cultural heritage” (Odeny, 2013: p. 4).

Based on the prevailing land relations, “most women remain dependent on the existence and goodwill of male relatives for access to land” (Allendorf, 2007).

It has therefore become important to “explore gender inequalities in the control of productive resources, together with policies and institutional processes underpinning gender inequalities in land” (Tsikata and Amanor-Wilks, 2009: p. 3). In the 2000s, “women’s rights to land have remained at the core of the quest for gender equality in Kenya” (Kameri-Mbote, 2009: p. 87). Although, there are no legal barriers on women’s property rights in Kenya as stipulated in the National Land Policy (Government of Kenya, 2009); however, this provision in practical terms, has yielded little success in respect to women’s ownership of land. This is due to several impediments, which are structural, economic and cultural in nature. Of all the limitations, the most complex to abolish is the cultural restraint to the attainment of women’s property rights.

## Methodology of Study

The article utilizes the qualitative research method through secondary and primary sources of data. Primary data were sourced from unstructured interviews (4 respondents) and focus group study (14 respondents). Respondents were purposively selected to engage the root causes of unequal access to land and unravel the linkages between women’s land rights and African cultural values. The respondents comprised of South Africans and Zambian nationals, Camerounians, Nigerians, Kenyans and Rwandans (who reside in South Africa). They were made up of officials of non-governmental organizations (KwaZulu-Natal Christian Council and Association for Rural Advancement involved in land reform advocacy in Southern Africa), 2 South African government agencies that deal with land reform, legal practitioners on women and property rights in Africa, academic experts in land reform and gender studies, women’s community leaders and others involved in land-related issues in their respective communities and countries (land-owners and the landless). In total 6 men and 8 women participated. The interviews were conducted in KwaZulu-Natal and Northern Cape Provinces from September to October 2017; while the focus group study was held in Pietermaritzburg, South Africa (October 2017). Respondents, stakeholders involved in the gender-land reform convergence, offered their perceptions on the vexing land crisis in Africa. The University of Zululand Research Ethics Committee approved the conduct of the research, and written informed consent was obtained from participants. The research was guided by the principle of anonymity.

## Land Reform in Africa: a Motivation

Across Africa, land-related conflict, gender-based land agitations, land hunger, illegal land occupations and dwindling land productivity are evidence of the need to rethink the existing land arrangements. Although post-colonial African states have had to embark on land reform, African populations, especially those in the rural areas, continue to face pressures on land resources. This speaks of a historical shift from pre-colonial land abundance to relative land inadequacy in several parts of the continent, spurring land reform. However, Whitehead and Tsikata noted that,

“African countries differ widely with respect to contemporary levels of land scarcity. In the context of an absolute rise in total populations, the severity of land scarcity depends on a country’s particular experiences of the colonial appropriation of land, of the commercial development of agriculture and the nature and degree of urbanization” (Whitehead and Tsikata, 2003: p. 68).

The issue of land, as manifested in countries like Zimbabwe, South Africa, Kenya, Ethiopia and Ghana, has become an important focal point for radical and democratic struggles, as land hunger and protracted land conflicts assumed an international character. The annexation of expanses of hectares of land for tourist enterprises and extraction points for mineral resources has curtailed indigenous population’s access to land in Africa (Whitehead and Tsikata, 2003).

Part of the main goals of land reform is to reduce poverty and address inequality. Unequal land relations lead to unequal opportunity. Land reform ‘matters’ because of its effect on poor people (Lipton, 2009: p. 1). Furthermore, “land is an especially suitable asset to try and get distributed more equally in developing countries, because it is particularly likely to be ascribed rather than earned” (Lipton, 2009: p. 22). For instance, South Africa has one of the most unequal income distributions in the world and skewed land arrangements have a role to play. As posited by Whiteford and McGrath (cited in Lahiff, 2003), income and material quality of life are strongly linked with race, location and gender. additionally, land inequality is also a strong determinant of livelihood, especially for those in the rural areas. Land is poor people’s main productive asset and a vital resource, which has socio-economic, political, environmental, cultural and development significance (Moyo, 2003; Kameri-Mbote, 2009; Lipton, 2009; Akinola, 2016, 2018).

The land distortions, dispossessions and inequality that Africa had experienced under colonial and Apartheid regimes, respectively, have imposed the responsibilities on post-colonial African states toward reforming the land sector. The “struggles over land ownership and land alienation, date back to the colonial period. The colonial states perpetuated and sometimes imposed patriarchal structures of land use and ownership within ‘native’ communities and reinforced a gender division of labor, which empowered men and disempowered women” (Mbilinyi and Shechambo, 2009: p. 96). It is important to acknowledge the intrinsic value of women’s empowerment and gender equality in the eradication of poverty and hunger, and improvement in food security (Malapit and Quisumbing, 2015: p. 54). One of the motivations for the quest toward the institutionalization of women’s rights is the empowerment and welfare argument. Proponents of the empowerment perspective regard women’s land rights as an important instrument to empower women for driving the development goal of the state in question (Allendorf, 2007). A research conducted in Asia confirmed that women in Thailand and India, who owned land, had more domestic economic power than those who did not own land (Allendorf, 2007). The welfare argument holds that the promotion of women’s land rights would enhance women’s welfare and well-being, their families,’ and that of the broader community. According to Allendorf (2007), the welfare argument “rests on the notion that resources put in the hands of women, rather than men, are more likely to be used to the benefit of children and others.”

Odeny (2013) reiterates the central place of women’s land rights in developmental discourse because of its impact on women’s access to land resources, security of the land tenure system, agricultural productivity and improvement in livelihood. However, women’s contributions to developmental initiatives are impeded by both socio-economic and cultural variables. Women have restricted access to credit facilities and lack the financial wherewithal to purchase land, more so in the capital-intensive and market economy (formal or informal land markets) that land has been subjected to in the country. This is the reality in Kenya and other African countries.

There are so many female-headed households, resulting from the demise of husbands, high rates of divorce, or the prevalence of ‘single motherhood’ in countries like South Africa, who are in need of land resources as economic and productive assets. According to Garvelink (2012), “widows are the most likely sector of society to lose their land and other assets, further disadvantaging them and their children.” The reality of land policy in the immediate post-colonial Africa, and a need to protect women who fall into these categories, as well as their dependents, explains the calls for reforming the existing land arrangements and tenure systems. Although there are other existing forms of land reform - nationalization or state-led reform, market-driven reform and radicalization of reform – a need for protecting women’s land rights, through land ownership and registration, has become challenging under the market-inclined and capital-intensive land reform scheme. As reinforced during the focus group study, land, which was then a communal asset, was entrusted in the hands of community or family leaderships, has been transformed into ‘controlled’ capital and economic objects under the modern state system. This was supported by Whitehead and Tsikata (2003: p. 70), “indigenous African land holding was viewed as ‘communal,’ and individual proprietary ownership was interpreted as a more developed form of land tenure linked to the development of market exchange.”

Some women who have been granted access to land in both rural and urban areas have struggled to obtain title deeds, while registration for such lands has proven to be very difficult. Land registration has become pertinent under the market-driven or liberal-driven land arrangements obtainable in post-colonial Africa. This was contrary to the pre-colonial land tenure system, where the communal use of, or access to, land was prevalent and land ownership was relegated to the background. For instance, a reform of the tenure system in South Africa has been mostly ignored. This raises more concern due to its high impact on other reform programs and a negative effect on rural populations, mostly women. In the South African context, tenure reform encompasses “the protection of the rights of residents of privately owned farms and state land, together with the reform of the system of communal tenure prevailing in the former homelands” (Lahiff, 2003: p. 41). In the country, “much of land reform policy can be seen as addressing the injustices of the past by returning land from the historically privileged to the historically oppressed” (Lahiff, 2003: p. 43). Both men and women have been ‘historically oppressed’; disposed of their land and habitation and restrained to the former homelands. The reform agenda of the government reinforced the patriarchal nature of the communal land system, to the detriment of the women who have shown evidence of ‘land-related injustices’ and a need for land resource.

## Culture and Women’s Land Rights: an Intersection

Stakeholders in the African land reform projects have encountered diverse barriers toward the equal application of land policies to both males and females, and this has been a clog in the wheel of land reform in Africa. In Africa, women own less than 1% of the land. Thus, the impact of land reform on rural women has been relatively insignificant in many parts of Africa as a result of the cultural limitations on women’s land rights. Both in the former-settler colonies in Southern and Eastern Africa, which have experienced land concentration and plantation agriculture, and West Africa as well with its limited producer-based production systems, women’s access to land had faced systemic deprivations (Tsikata and Amanor-Wilks, 2009). However, women in the settler colonies in Africa experienced less access and control over land than their counterparts in West Africa. Under African cultural heritage, and particularly, in Cameroon, women do not receive donations of land nor do they enjoy user rights to land from either husbands or fathers. Wives can only inherit land from husbands if they have male children who will later own the land; the mother only holds such land in trust for the male child/children (Wanyeki, 2003). In Nigeria, the Land Use Act of 1978 provides for gender-neutral land rights, but in reality, due to cultural affinity, women are mostly denied the rights to land. In some societies like Rwanda, under the *ubukonde* system, land was claimed by whoever first cultivated it. Therefore, land use or cultivation was regarded as land ownership (Wanyeki, 2003). This was corroborated by a respondent who insisted, “there was no such thing as individual land ownership; once you cultivate a barren land in the community, the land belongs to the individual. Ownership is not even static. The land may fall into another man’s hand if and when the land was no more in use for a sustained period.” Although,

“More practicable legislation and enforcement have reduced aspects of gender discrimination. However, land injustice to women-and the resulting vulnerability and inefficiency-are often culturally (and even religiously) deep-rooted, and highly localized. Practical law enforcement usually bends to such powerful, yet diffused, winds” (Lipton, 2009: p. 22).

Despite women’s high degree of involvement in agriculture, data from a nationally representative survey indicate that most agricultural parcels (85%) are owned exclusively by the individual male, (9.8%) by the individual female, and only (3.5%) jointly (Deere et al., 2012). In Africa, women in the urban areas tend to enjoy more property rights than those in the rural areas. Cultures and traditional norms tend to be more preserved among the closely knitted rural community than the cosmopolitan urban population. Several reasons explain this, among which are: the higher level of educated elites in the urban areas, monitoring of legal provisions by government institutions and an erosion of some cultural affinities in the cities due to their cosmopolitan nature. For instance, women residing in Ghanaian urban areas have a substantial advantage over rural women, owning 23% as opposed to 15% of couples’ wealth, respectively (Deere et al., 2013: p. 256).

One of the challenges of women’s access to their husbands’ properties and wealth is the structural obstacles associated with women’s registration of properties. In terms of obtaining a deed of land ownership, very few women have their land fully registered in Kenya, and many are sometimes subjected to male arm-twisting, especially within communal relations (Kameri-Mbote, 2009: p. 88). Within families, men are fond of selling family land without consulting their wives or female family members and every attempt for the women to push for the rights for joint-ownership (husband and wives, or male and female) of land has been unachievable. In Kenya, registration of land,

“Was bound to exclude most women from acquiring titles to land since they only had use rights, while men retained those of ownership and allocation, and the tenure reform process only considered people with ownership rights. In most cases, families designated one member, usually the eldest son or the male head of the household, to be registered as the absolute owner without realizing the latitude that person would have” (Kameri-Mbote, 2009: p. 88).

In other societies of developing countries, women do not have direct claim to land. The exercise of their claims to properties are subsumed in their husband’s rights (Jacobs, 2002: p. 888), or that of their sons. A respondent from South Africa recalled an experience,

“My mother was denied the right to inherit the family land after the demise of my father. The family head told her to forget about her demand for landed property because she is a woman. She tried to get land through the redistributive and restitution program of the South African state, but all proved abortive. She was later advised to apply through my brother. It was only at this point that a small parcel of land was allocated to us through my brother.”

This point was also buttressed by a respondent from Nigeria, who recalled that her sister suffered a similar fate in her bid to access family land.

In Africa, the customary land tenure system is driven by linage or clan control. The land devolves to the son in patrilineal systems and to the sister’s son in matrilineal systems (Ensminger, 1997: p. 169). Societies continue to give preference to sons over daughters, men over women, as custodians of land and other properties. Indeed, where laws that promote gender parity have been made, traditional practices are mostly biased against women’s land inheritance. These cultural limitations to the actualization of women’s rights (like the principles of hereditary property and patrimony) evolved from indigenous law and traditional practices. Despite the modernization of politics and a shift to liberal political ideology, states and societies in Africa continue to resist gender-free property rights.

Another factor impeding land tenure reform is the position of African traditional elites in the land reform project. This was unanimously raised during the focus group study. In many parts of Africa, traditional authority has attained custodianship of culture and land and become ‘land distributor and land entrepreneurs’. They link the present with the past, and therefore espouse the cultural values of the pre-colonial African heritage: an era shrouded in extreme denials of women’s land claims. In some instances, land governance rests with the traditional councils. There is no gender parity in the composition of the traditional council that redistributes the land. The traditional authorities adhere to a sustained principle of hereditary rule, which favors patriarchal system. Moreover, the principle of equality is alien to customary law which regulates the conduct of the traditional council (Rangan and Gilmartin, 2002: p. 638). The same male domination is recorded in states’ institutions those in charge of land reform are no exception.

African culture acknowledges the property rights of men or men-dominated kinship groups, thereby limiting the ability of women to claim, own or inherit land (Garvelink, 2012). Customary law is one of the foundations on which property ownership rests. According to Eniola and Akinola (2019), “the social legitimacy of these traditions is a stumbling block in realizing women’s property rights as these traditions regard women as being incapable of exercising control over landed property.” Based on this principle, devolution of property is patrilineal. Land ownership “follows the blood line and is based on the belief that men as permanent members of the family will perpetuate the father’s dynasty while women are expected to marry and cease to be members of their father’s family’ (Eniola and Akinola, 2019). As revealed by Garvelink (2012), “while statutory law may be gender neutral, customary law prevails and is based on a patriarchal system.” Men’s domination is not only prevalent within cultural institutions, African cultural values reinforce the domination of men in politics, and in key decision-making structures in the communities and families. Men determine the course of politics. They are vital in economic decisions and are the custodians of properties (land inclusive); hence, “land-related gender inequalities are culturally created” (Yeboah, 2014). Moreover, their property rights under communal ownerships and ranches (groups established in pastoral areas to own land) are not properly defined, and are thus complex to attain. These have reinforced the low economic status of women compared to that of men, and the struggle to wrestle the important institutions that deal with land ownership, access and land tenure system from men has been met with stiff resistance by the men. In some cases, a section of the women has supported the gender gap in the cause of maintaining social stability and in defense of African traditional values.

One of the appeals for the adoption of nationalization policy in the land sector or state-led reform was the utility of the reform agenda by political elites for political patronage (Mamdani, 1996). In many parts of Africa, especially in the former-settler colonies like South Africa and Zimbabwe, land, seen not only as a tool to redress historical injustices or redistribute wealth, has also become a political tool for mass mobilization during transition or a mechanism for regime consolidation. At these periods, state actors engage in rhetoric to appease the electorate by making high-sounding gender-neutral comments on land. This was necessary to garner votes from the women. Despite the recent ascendency of women’s involvement in the electioneering process and politics in general, a greater percentage of women are still located outside the political orbit. Women’s low level of participation in politics and decision-making processes continues to curtail their property rights. Women are excluded from decisions on the allocation and transfer of land in their various communities (Kaarhus et al., 2005: p. 482). Inequality in the rights to land promotes women’s socio-political subordination and poverty (Razavi, 2003: p. 4). Whitehead and Tsikata (2003) commented in a similar vein, “the main problem is that women have too little political voice at all the decision-making levels that are implied by the land question: at local-level management systems, within the formal law and also within the government and civil society itself”. The recent global push for women’s empowerment and participation in politics (in countries like Nigeria, Kenya, Rwanda, and South Africa) has increased the number of women in key decision-making institutions in Africa, but the domineering stance of the men has been unabated.

African traditional communities still conceive the duty of a woman to be primarily that of child bearing and rearing. Almost all the respondents lent credence to this fact and recalled their own experiences of restricted roles to the ‘homes and kitchen’ in their respective families and communities. African culture frowns on women’s participation in economic activities for survival: that role falls unto the man or husband, who is expected to provide for the woman and his entire family (Eniola and Akinola, 2019). Women, seen as adjuncts to their husbands, are not expected to own land or claim the produce thereof. This is borne out of the traditional belief that the land, the produce of the land and the woman, all belong to the husband. Women are seen as properties, under the ownership of the man. Furthermore, at the demise of the husbands, the wives are not expected to inherit their husbands’ properties, since they are part of the properties that would be inherited by the husband’s family. It is prevalent in many parts of Nigeria to eject a widow from the husband’s property. The status of a woman continues to change due to their growing involvement in productive endeavors and the globally inspired efforts at women’s empowerment. Women are now farmers and could engage in several economic activities in support of their husbands. For instance, in a rural community of KwaZulu-Natal Province (South Africa), a respondent said, “women that are married and widowed have access to their husbands’ and late husbands’ land, respectively, but unmarried women and in some cases, widows that have no male child or children are denied access.” Generally, the rights to own vital properties such as land still remains denied in many rural Africa. Lack of the required access to land has aggravated the endemic land underutilization and low agricultural productivity in Africa.

## Women, Land Productivity and Agricultural Development

There are divergent motivations for the increased agitations for land rights in Africa, but the most prevalent driving force is for agricultural purposes. This could be in form of farm labor and peasant or commercial farming. In Africa, women have somehow managed to amass the highest agricultural labor-force participation rates in the world and have about 50% rates in Africa (Food and Agriculture Organization [Fao], 2011). For instance, women and girls are active in Ghana’s agricultural sector: females accounted for 49% of the economically active population in 2010, of which agriculture employed 49.3% (Food and Agriculture Organization [Fao], 2011). Accordingly,

“Most rural African women play a substantial part in primary agricultural production, making the complex of local norms, customary practices, statutory instruments and laws that affect their access to and interests in land very significant (not only to them, their dependents and their male relatives, but also arguably to levels of agricultural production)” (Whitehead and Tsikata, 2003: p. 68).

The special place of agriculture in states’ quest for development was noted by FAO, which posits thus,

“Governments, donors and development practitioners now recognize that agriculture is central to economic growth and food security – particularly in countries where a significant share of the population depends on the sector – but their commitment to gender equality in agriculture is less robust” (Food and Agriculture Organization [Fao], 2011: p. 3).

The fact remains, without land there is no agriculture.

Land acquisition, reforms and resettlement schemes in Africa have reduced women’s rights to hold land for subsistence farming. Therefore, “given women’s crucial role in food production and provision, any set of strategies for sustainable food security must address their limited access to productive resources” (Kaarhus et al., 2005: p. 482). Women continue to contribute significantly to food production through farm labor, food processing and storage, and transportation of agricultural products (Ilumoka, 2012: p. 423). However, while women’s subsistence farming plays an important role in ensuring food security, there have been some challenges in the attainment of optimal productivity. Denial of access to land, due to cultural restraints and patriarchal land tenure systems in most African states, have limited women’s efficiency in agriculture (Razavi, 2003: p. 4). A report reiterates the significance of women in agriculture. It maintains that,

“Closing the gender gap in agriculture would generate significant gains for the agriculture sector and for society. If women had the same access to productive resources as men, they could increase yields on their farms by 20–30 percent. This could raise total agricultural output in developing countries by 2.5–4 percent, which could in turn reduce the number of hungry people in the world by 12–17 percent” (Food and Agriculture Organization [Fao], 2011: p. 5).

The development of capitalism and capitalization of the agricultural sector has engendered contradictions that negatively impacted on women’s livelihood and impeded gender parity in land-labor relations. As noted by Tsikatal (2009), accumulation of land due to sustained promotion of capitalist agriculture led to pervasive landlessness among agricultural laborers, who had no time, support or motivation to engage in peasant farming. Women in this category are seldom poorly remunerated, experiencing extensive work-hours, poor conditions of labor, uncertain social protection, and exposure to diverse health challenges. One of the objectives of land reform is to reverse the trend. However, despite the implementation of land reform in many African states, women are still subjected to poor labor relations, particularly in rural areas.

African states have ignored the interconnectedness of labor and land relations. There is a need to draw linkages between land and labor relations in Africa and examine cases of discrimination in remuneration of both genders of those who work the land, which has impacted negatively on the livelihoods of women, especially the unmarried and widowed. As revealed in Tsikatal (2009: p. 13), “studies have demonstrated how inequalities between men and women in the ownership, control of and access to land have resulted in gender inequalities in their livelihood outcomes.” Furthermore,

“The importance of land and labor rights to women in sub-Saharan Africa is on account of the predominantly agrarian nature of livelihood activities, whose low technological base makes labor a critical factor. Beyond agriculture, land has a wide array of uses in the organization of livelihoods and is also the basis of social and political power, and therefore at the heart of gender inequalities in the control of resources” (Tsikata and Amanor-Wilks, 2009).

Though there are few cases of discrimination in the wages of farm labor along gender divides and women receive less pay than their men counterparts in some societies in Nigeria and rural South Africa, Africa has scored high on gender parity in farm labor wages. A gender-free remuneration system would increase the standard of living of women and their participation in agriculture. Also, “increasing poor people’s share of land rights, especially perhaps via land reform, can raise the poor’s income in five ways: via farm labor, land, and enterprise; via non-farm activity; and through economy-wide effects on growth and distribution” (Tsikata and Amanor-Wilks, 2009: p. 2).

In many African countries, land reform focuses more attention on the allocation of land to women and securing their land rights toward improved livelihood through farming (either peasant or large-scale), but less attention has been paid to the way unfavorable labor practices or discrimination against women in respect of farm labor have further impoverished the women, thereby restricting the gains of land reform. Poor people depend on labor for most of their incomes, and small farms use more labor per hectare than large mechanized farming. Thus, an effective agrarian reform would allocate farms into smaller farm units, raise labor demand, and – directly or indirectly – increase the labor income of farm workers. Women are mostly engaged in farm labor in most African countries. Cameroon is an exception: women are more involved in farming than men.

## Conclusion

As revealed in the article, African cultural practices are not in tandem with modern socio-economic realities. Societies have undergone changes, human beings are dynamic; thus, there should be room for the transformation of cultures to accommodate the realities of a changed world. African states, which desire development under the framework of liberalism, should be alive to their responsibilities. Since African governments have shown a willingness to enact gender-free laws, they must demonstrate renewed efforts toward enforcing it, and reform the social and political institutions that deal with land issues. As revealed by a respondent, “the world has become a fast-changing world, laws should respond to this dynamism, without complete disbursement of core African values in relation to the position of women as care-givers in the home, but more focus should be directed at abolishing institutional or cultural impediments to women empowerment.” Thus, actors in the women’s property rights scheme should dialog with states’ institutions and traditional authorities toward addressing women’s land rights and also ensuring that adherence to cultural practices in the rural areas does not contradict constitutionalism.

Democratization and devolution of powers, with its attendant resource re-allocation and protection of women’s property rights remains the sure path to development of the African states and societies under the liberal political and economic frameworks. Gender-parity in the area of land resource should be located in the prevailing state-centric land policy. Many of the respondents held that a complete return to customary laws guiding land or recourse to traditional land governance would serve the interest of the male and consolidate the patriarchal nature of resource allocation in African communities. As canvassed by the FAO Director-General, Jacques Diouf, “women must be seen as equal partners in sustainable development. Achieving gender equality and empowering women is not only the right thing to do; it is also crucial for agricultural development and food security” (Food and Agriculture Organization [Fao], 2011: p. vii). The germane roles of women in the agricultural sector should be recognized and enhanced, especially by the antagonists of women’s land rights. As reported by a respondent from Cameroon, “in my country, women are decisive actors in the agricultural sector.” No doubt, it is challenging to achieve gender reform side-by-side with land reform. Land reform, without an effective policy on gender equality, is not likely to increase women’s rights to land in highly patriarchal societies found in Africa and elsewhere.

There are other steps to be taken by governments to complement land reform and the legality of women land rights. One of such is the integration of women into decisive institutions in the land sector and other areas of women’s empowerment. The attainment of women rights goes beyond legality or creation of institutions of land. For example, in Kenya, The National Land Policy, adopted in 2009, reinforced the basis for the actualization of women’s land rights, but its implementation has been restricted by societal norms and traditional practices that have strategically elevated men as the custodians of land and other sensitive matters in the family and, by extension, in the community. Thus, attainment of women’s land rights should not only be subjected to the drafting of laws, but the need for a change of Africa’s traditional belief systems is at the core of the enhancement of women’s land rights in Kenya and Africa in general. Just like a respondent echoed, “it is important to reform the reformers.” He further posits that the drivers of the reform agenda are mainly men, who are receptive to change in gender relations. Their ‘sympathy’ for an African culture that denies women access to productive assets, land, should be replaced by accepting the women as partners in the quest to sustain agricultural production and productively utilize the large parcels of land that remain fallow. This should not be seen as ‘blank’ or ‘mindless’ calls for the allocation of land to women (just because they are women), but land allocation should not be driven by gender considerations or other forms of primordial factors.

## Ethics Statement

The study was conducted in accordance with the University of Zululand Ethical considerations. The “University of Zululand Research Ethic Committee” (UZREC) gave ethical approval for the conduct of the field study and categorized it under medium risk. The certificate number is UZRECC 171110-030 Dept 2017/141. All subjects gave informed consent to participate.

## Author Contributions

The author confirms being the sole contributor of this work and has approved it for publication.

## Conflict of Interest Statement

The author declares that the research was conducted in the absence of any commercial or financial relationships that could be construed as a potential conflict of interest.

## Footnotes

1 Land reform is the change in existing land patterns and policy within a geographically defined territory. This could be toward creating more access to land for vulnerable groups, making the state the custodian of land, redistribution of land to other users, ensuring tenure security or correcting land-related injustice in the society.

2 Land rights are legitimate entitlements to land and the associated benefits derivable from the land.

## References

[B1] Akinola A. O. (2016). Human rights, civil society and the contradictions of land reform in South Africa. *Politeia* 35 52–70. 10.25159/0256-8845/2365

[B2] Akinola A. O. (2018). South African land reform: an appraisal. *Afr. Rev.* 10 1–16. 10.1080/09744053.2017.1399560

[B3] Allendorf K. (2007). Do women’s land rights promote empowerment and child health in Nepal? *World Dev.* 35 1975–1988. 23700354 10.1016/j.worlddev.2006.12.005 PMC3657746

[B4] Deere C. D. Oduro A. Swaminathan H. Doss C. R. (2012). “Property rights and the gender distribution of wealth in ecuador, Ghana, and India,” in *Proceedings of the Gender Asset Gap Project Working Paper Series No. 13 Centre of Public Policy* (Bangalore: Indian Institute of Management Bangalore).

[B5] Deere C. D. Oduro A. Swaminathan H. Doss C. R. (2013). Property rights and the gender distribution of wealth in ecuador, Ghana and India. *J. Econ. Inequal.* 11 249–265. 10.1007/s10888-013-9241-z

[B6] Eniola B. Akinola A. O. (2019). “Women rights and land reform in Africa: Nigeria and South Africa in comparison,” in *The Trajectory of Land Reform in Post-Colonial African States: The Quest for Sustainable Development and Utilization* eds Akinola A. O. Wissink H. (Cham: Springer International Publishing).

[B7] Ensminger J. (1997). “Changing property rights: reconciling formal and informal rights to land in Africa,” in *The Frontiers of the New Institutional Economics*, eds Drobak J. N. Nye J. V. C. (San Diego, CA: Academic Press) 165–196.

[B8] Food and Agriculture Organization [Fao]. (2011). *The State of Food and Agriculture 2010–2011. Women in Agriculture: Closing the Gender Gap for Development.* Rome: Food and Agriculture Organization of the United Nations.

[B9] Garvelink W. (2012). *Land Tenure, Property Rights, and Rural Economic Development in Africa.* Washington, DC: Center for Strategic and International Studies.

[B10] Government of Kenya. (2009). *National Land Policy.* Nairobi: Government Printer.

[B11] Ilumoka A. (2012). Globalisation and the re-establishment of women’s land rights in Nigeria: the role of legal history. *Chic. Kent Law Rev.* 87 423–437.

[B12] Jacobs S. (2002). Land reform: still a goal worth pursuing for rural women? *J. Int. Dev.* 14 887–898. 10.1002/jid.934

[B13] Kaarhus R. Benjaminsen T. Hellum A. Ikdahl I. (2005). Women’s land rights in Tanzania and South Africa: a human rights based perspective on formalisation. *Forum Dev. Stud.* 32 443–482. 10.1080/08039410.2005.9666323

[B14] Kameri-Mbote P. (2009). What would it take to realise the promises? Protecting women’s rights in the Kenya national land policy of 2009. *Fem. Afr.* 12 81–94.

[B15] Lahiff E. (2003). *The Politics of Land Reform in Southern Africa. Sustainable Livelihoods in Southern Africa. Research Paper 19.* Brighton: Institute of Development Studies.

[B16] Lipton Michael. (2009). *Land Reform in Developing Countries: Property Rights and Property Wrongs.* New York, NY: Routledge: Tailor & Francis.

[B17] Malapit H. J. Quisumbing A. R. (2015). What dimensions of women’s empowerment in agriculture matter for nutrition in Ghana? *Food Policy* 52 54–63. 10.1016/j.foodpol.2015.02.003

[B18] Mamdani M. (1996). *Citizen and Subject: Contemporary Africa and the Legacy of Late Colonialism.* Princeton, NJ: Princeton University Press.

[B19] Mbilinyi M. Shechambo G. (2009). Struggles over land reform in Tanzania: experiences of Tanzania gender networking programme and feminist activist coalition. *Fem. Afr.* 12 94–104.

[B20] Moyo S. (2003). The land question in Africa: research perspectives and questions. *Draft Paper Presented at CODESRIA Conferences on Land Reform, the Agrarian Question and Nationalism in Gaborone* Dakar.

[B21] Odeny M. (2013). Improving access to land and strengthening women’s land rights in Africa. *Paper Prepared for Presentation at the Annual World Bank Conference on Land and Poverty* (Washington, DC: The World Bank).

[B22] Rangan H. Gilmartin M. (2002). Gender, traditional authority, and the politics of rural reform in South Africa. *Dev. Chang.* 33 633–658. 10.1111/1467-7660.00273

[B23] Razavi S. (2003). Introduction: agrarian change, gender and land rights. *J. Agrarian Chang.* 3 2–32. 10.1111/1471-0366.00049

[B24] Tsikata D. Amanor-Wilks D. (2009). Editorial: land and labour in gendered livelihood trajectories. *Fem. Afr.* 12 1–10.

[B25] Tsikatal D. (2009). Gender, land and labour relations and livelihoods in sub-saharan Africa in the era of economic liberalisation: towards a research agenda. *Fem. Afr.* 12 11–30.

[B26] United Nations Development Programme [UNDP]. (2015). *The Millennium Development Goals Report 2015. 06 July.* http://www.undp.org/content/undp/en/home/librarypage/mdg/the-millennium-development-goals-report-2015.html

[B27] Wanyeki L. M. (2003). *Women and Land in Africa: Culture, Religion and Realizing Women’s Rights.* New York, NY: Zed Books Ltd.

[B28] Whitehead A. Tsikata D. (2003). Policy discourses on women’s land rights in Sub-Saharan Africa: the implications of the re-turn to the customary. *J. Agrarian Chang.* 3 67–112. 10.1111/1471-0366.00051

[B29] Yeboah E. (2014). *Women’s Land Rights and Africa’s Development Conundrum – Which Way Forward? IEED, International Institute for Environment and Development.* http://www.iied.org/womens-land-rights-africas-development-conundrum-which-way-forward

